# Immunogenicity and safety of a combined DTPa-IPV/Hib vaccine administered as a three-dose primary vaccination course and a booster dose in healthy children in Russia: a phase III, non-randomized, open-label study

**DOI:** 10.1080/21645515.2020.1720437

**Published:** 2020-02-12

**Authors:** Victor Romanenko, Irina Osipova, Anna Galustyan, Michael Scherbakov, Nathalie Baudson, Déborah Farhi, Luis Anaya, Sherine O. Kuriyakose, Nadia Meyer, Winnie Janssens

**Affiliations:** a Children’s City Hospital №11, Ekaterinburg, Russian Federation; b OOO “ASKO-MED-PLUS”, Barnaul, Russian Federation; c Medical Technologies Ltd, St. Petersburg, Russian Federation; d GSK, Moscow, Russian Federation; e GSK, Rixensart, Belgium; f GSK, Wavre, Belgium; g GSK Asia Private Ltd., Bengaluru, India

**Keywords:** DTPa-IPV/Hib, acellular pertussis, diphtheria, tetanus, poliovirus *Haemophilus influenzae* type b, pediatric population, immunogenicity, safety, reactogenicity

## Abstract

We assessed the immunogenicity and safety of the combined diphtheria-tetanus-acellular pertussis-inactivated poliovirus/*Haemophilus influenzae* type b vaccine (DTPa-IPV/Hib) in children in Russian Federation aiming to support the registration of the vaccine in Russia. In this phase 3, non-randomized, open-label study (NCT02858440), healthy children received three primary doses at 3, 4.5, and 6 months of age (N = 235) and a booster dose at 18 months of age (N = 225). Seroprotection rates against diphtheria, tetanus, Hib, and poliovirus 1–3, seropositivity rates against pertussis antigens, and antibody geometric mean concentrations/titers for all antigens were evaluated one month post-primary and post-booster vaccinations. Solicited local and general adverse events (AEs) were collected during a 4-day period and unsolicited AEs during a 31-day period post-vaccination. Serious AEs were recorded throughout the study. At post-primary vaccination, all infants were seroprotected against diphtheria, tetanus, and poliovirus 1 and 2, 99.3% against poliovirus 3, and 98.4% against Hib. At least 98.9% of participants were seropositive for the three pertussis antigens. At post-booster vaccination, all toddlers were seroprotected/seropositive against all vaccine components. The most frequent local and general solicited AEs were redness, reported for 52.6% and 44.9% of children, and irritability, reported for 64.7% and 39.1% of children, post-primary and post-booster vaccination, respectively. Unsolicited AEs were reported for 20.4% (post-primary) and 5.8% of children (post-booster vaccination). Most AEs were mild or moderate in intensity. Six serious AEs were reported in three (0.4%) children; none were fatal or assessed as vaccination-related. DTPa-IPV/Hib proved immunogenic and well tolerated in the Russian pediatric population.

## Introduction

Although routine infant vaccination significantly decreased the morbidity and mortality associated with previously common childhood infectious diseases, including diphtheria, tetanus, pertussis, *Haemophilus influenzae* type b (Hib) and poliomyelitis, disease burden remains substantial, affecting populations worldwide.^1,2,3,4,5–6^ The crowded childhood immunization routine schedules might be a potential deterrent for parents and providers to comply with recommendations and this can result in decreased vaccine coverage, and ultimately, disease outbreak.^7^ Introducing combination vaccines to replace complex immunization schedules has several benefits, such as ease of storage, simplified administration, fewer injections, increased patient and health care acceptance, higher rates of compliance to vaccination schedules, improved coverage rates, reduced shipping and administration costs, reduced confusion over labeling in the medical office, and reduced number of visits.^8,9–11^

A pentavalent diphtheria-tetanus-acellular pertussis-inactivated polio and Hib conjugate vaccine (DTPa-IPV/Hib; *Infanrix-IPV/Hib*, GSK) has been widely used in several countries across the world since its first license in 1997. The vaccine was shown to be immunogenic, with an acceptable safety profile, when administered as primary and/or booster vaccination according to different schedules.^12–17^ In the Russian Federation, a 3-dose primary vaccination schedule (with doses administered at 3, 4.5, and 6 months of age), and a booster dose at 18 months of age are currently recommended against the following diseases: diphtheria, tetanus, pertussis, poliomyelitis and diseases caused by Hib.^18,19^ DTPa-IPV/Hib combines all antigens in one single formulation and its use can therefore complement the current standard of care for hepatitis B immunization in Russian children.

The aim of the present study was to evaluate the immunogenicity and safety of the combined DTPa-IPV/Hib vaccine when administered as a 3-dose primary vaccination course at 3, 4.5, and 6 months of age and as a booster dose at 18 months of age in healthy children according to the Russian immunization schedule to support the registration of the combination vaccine in this country.

## Methods

### Study design and participants

This phase 3, single group, non-randomized, open-label study was conducted in five centers in the Russian Federation between September 2016 and November 2018. Healthy infants, born full-term, aged 3–4 months (90–120 days) at the time of the first vaccination, for whom written informed consent was obtained from their parents/adoptive parents were enrolled in the study. Infants were not eligible if they had received immunosuppressant or immune-modifying drugs or previous DTP, poliovirus or Hib vaccination. A full list of exclusion criteria is provided in the Supplementary text.

Participants received four doses of combined DTPa-IPV/Hib as a 3-dose primary vaccination course at 3, 4.5 and 6 months of age and a booster dose at 18 months of age. At each vaccination, a 0.5 mL dose was administered intramuscularly in the upper side of the thigh.

An internet-based central randomization system was used to allocate treatment numbers by dose and to track enrollment in the study. Laboratory personnel performing sample testing were blinded to the treatment allocation.

In total, three commercial lots of both DTPa-IPV (liquid) and Hib (lyophilized) components were used in the study. Each dose of DTPa-IPV/Hib contained ≥30 International Units (IU) diphtheria toxoid, ≥40 IU tetanus toxoid, 25 μg pertussis toxoid (PT), 25 μg filamentous hemagglutinin (FHA), 8 μg pertactin (PRN), 40 D-antigen units (DU) inactivated poliovirus type 1, 8 DU inactivated poliovirus type 2, 32 DU inactivated poliovirus type 3, 10 μg purified Hib polyribosyl-ribitol phosphate (PRP) capsular polysaccharide conjugated to tetanus toxoid (~25 μg), and 500 µg aluminum hydroxide as adjuvant. Co-administration of a vaccine against hepatitis B virus (HBV) and other vaccines given as part of the national immunization schedule and as part of routine vaccination practice were allowed at any time during the study period.

The study was conducted in compliance with the Declaration of Helsinki, the International Conference on Harmonization Guideline for Good Clinical Practice, and all applicable local regulations. The study protocol and informed consent were reviewed and approved by Independent Ethics Committees/Institutional Review Boards at each center. The trial is registered at http://www.clinicaltrials.gov (NCT02858440) and the full protocol is available at http://www.gsk-clinicalstudyregister.com/study/4677.

### Study objectives

The primary objective was to evaluate the immune responses to the vaccine components in terms of seroprotection rates for diphtheria, tetanus, Hib, and poliovirus serotypes 1–3 antigens, and in terms of seropositivity rates for pertussis antigens in infants one month after the third dose of the primary vaccination.

Secondary objectives were to assess the immune responses to the vaccine components in terms of seroprotection rates for diphtheria, tetanus, Hib, and poliovirus serotypes 1–3 antigens, and in terms of seropositivity rates for pertussis antigens in toddlers one month after the booster vaccination; antibody concentrations or titers against diphtheria, tetanus, Hib, poliovirus types 1–3, and pertussis antigens in children one month after both primary and booster vaccinations; as well as to evaluate vaccine safety and reactogenicity.

### Immunogenicity assessments

Blood samples (3.5 mL) were collected one month after the third dose of the primary vaccination and one month after the booster vaccination. Antibodies against diphtheria,^20^ tetanus,^21^ Hib PRP, and pertussis components^22,23^ were measured using standard in-house enzyme-linked immunosorbent assays. 96-well microplate coated with the corresponding purified antigen were incubated with dilutions of serum samples, controls, and standard. Microplate were washed and mouse horseradish peroxidase (HRP)-conjugated anti-human IgG monoclonal antibodies (diphtheria, tetanus, pertussis) or goat HRP-conjugated anti-human Ig polyclonal antibodies (Hib) were added. Enzyme activity was revealed spectrophotometrically using tetramethylbenzidine. Concentrations were calculated from the reference standard curve using a four parameters logistic fitting algorithm and expressed in IU/mL (diphtheria, tetanus, pertussis) or microgram (µg)/mL. Assay cutoffs (equals to the lower limit of precision and linearity) were 0.057 IU/mL (diphtheria), 0.043 IU/mL (tetanus), 0.066 µg/mL (anti-PRP), 2.693 IU/mL (PT), 2.046 IU/mL (FHA), and 2.187 IU/mL (PRN). Antibodies against poliovirus 1–3 antigens were measured by a standard in-house neutralizing antibody assay adapted from the WHO Guidelines for WHO/EPI Collaborative Studies on Poliomyelitis.^24^ All analyses were performed at the Clinical Laboratory Sciences (GSK, Rixensart or Wavre, Belgium) applying validated laboratory tests.

Seroprotection was defined as antibody concentrations ≥0.1 IU/mL for diphtheria and tetanus, ≥0.15 µg/mL (indicative of short-term protection) and 1.0 µg/mL (indicative of long-term protection) for PRP, and antibody titers ≥8 ED_50_ (titers expressed in terms of the reciprocal of the dilution resulting in 50% inhibition; samples with a titer greater than or equal to 1:8 is considered seroprotective) for poliovirus types 1–3.^25-27^ A generally accepted correlate of protection for *Bordetella pertussis* is not yet established since not only PT antibodies play an important role, but also other antibodies, such as FHA and PRN, as well as cellular immune responses seem to contribute to protection.^26,27^ In this study, participants with anti-PT, anti-FHA, and anti-PRN antibody concentrations above the assay cutoffs were considered seropositive.

Antibody geometric mean concentrations (GMCs) and geometric mean titers (GMTs), and seroprotection/seropositivity rates were calculated one month following both primary and booster vaccination.

### Safety and reactogenicity assessments

Participants were observed for at least 30 minutes following the administration of the study vaccine for any immediate reactions. Solicited local (injection site pain, redness, swelling) and general (drowsiness, fever, irritability, loss of appetite) adverse events (AEs) occurring within the 4-day (days 0–3) period and unsolicited AEs occurring within the 31-day (days 0–30) period after each vaccine dose administration were recorded on diary cards by the participants’ parents/adoptive parents. All solicited local AEs were considered as related to vaccination. The causality of other AEs was assessed by the investigator. The intensity of all AEs was evaluated on a 3-grade scale from mild to severe. Severe (grade 3) AEs were defined as crying when a limb is moved (for pain), diameter >20 mm (for redness and swelling), axillary temperature >39.0°C (for fever), preventing normal everyday activities (for irritability and drowsiness), and as not eating at all (for loss of appetite). Large injection site reactions (swelling with a diameter >50 mm, noticeable diffuse swelling or noticeable increase of limb circumference) were recorded for up to 4 days (days 0–3) after the booster vaccination. Related and medically-attended AEs were also recorded. Serious AEs (SAEs) were collected during the entire study period. All unsolicited AEs and SAEs were classified using the Medical Dictionary for Regulatory Activities (MedDRA) Primary System Organ Class and Preferred Terms.^28^

### Statistical analyses

A sample size of 200 evaluable infants was requested by the local regulatory authorities; assuming a 15% drop-out rate, a total of approximately 235 infants were to be enrolled in the study.

For each vaccination course (primary and booster), immunogenicity analyses were performed on the according-to-protocol (ATP) cohort for immunogenicity, which included all vaccinated participants who met all eligibility criteria, complied with the protocol, and for whom assay results were available post-vaccination for at least one study vaccine antigen. Seroprotection rates, seropositivity rates, and antibody GMCs and GMTs were calculated with 95% confidence intervals (CIs) one month after the primary and booster vaccinations. GMC/GMT calculations were performed by taking the anti-log of the mean of the log_10_ concentration/titer transformations. Antibody concentrations/titers below the cutoff of the assay were given an arbitrary value of half the cutoff.

For each vaccination course, safety analyses were performed on the total vaccinated cohort, which included all participants who received at least one dose of the study vaccine. The percentage of infants with at least one solicited (local and general) and unsolicited AE was calculated after each vaccine dose and overall per participant, with exact 95% CIs.

## Results

### Demographics

**Figure 1.**
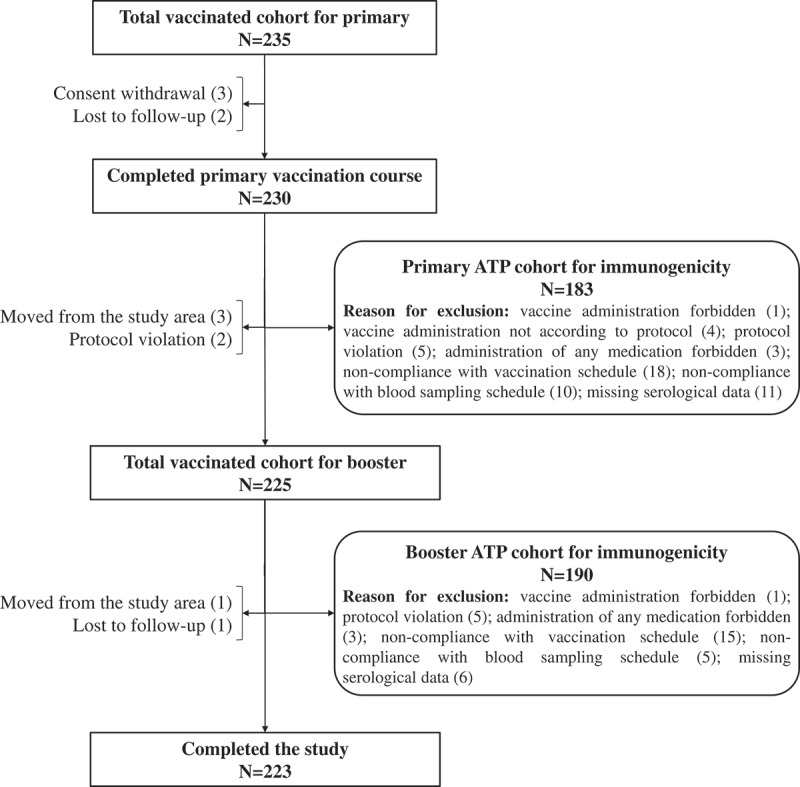
Flow of participants N, number of participants; ATP, according-to-protocol. Note: participants may have more than one reason for exclusion.

In total, 235 children were enrolled and vaccinated with three primary doses; 225 of them received the booster dose (Figure 1). The ATP cohort of the primary and booster vaccination courses included 183 and 190 participants, respectively. Reasons for exclusion from the ATP cohort, as well as reasons for withdrawal from the study are presented in Figure 1. The mean age was 14.1 weeks at the receipt of the first primary dose and 17.7 months at booster dose. All participants were of European heritage (Table 1). Vaccination against HBV was documented for 162 out of the 235 children, 115 of them received the vaccine at the same time as the study vaccine.

**Table 1.** Summary of demographic characteristics.

	Primary vaccination		Booster vaccination	
	TVC	ATP	TVC	ATP
N	235	183	225	190
Mean age at first dose (±SD), weeks/months*	14.1 ± 1.2	14.1 ± 1.2	17.8 ± 0.5	17.7 ± 0.4
Male, n (%)	124 (52.8)	94 (51.4)	118 (52.4)	101 (53.2)
White-Caucasian/European heritage, n (%)	235 (100)	183 (100)	225 (100)	190 (100)

TVC, total vaccinated cohort; N, number of participants; ATP, according to protocol; SD, standard deviation; n (%), number (percentage) of children in a given category. *Mean age expressed in weeks for primary vaccination and months for booster vaccination.

### Immunogenicity

One month after the third dose of primary vaccination, all infants had antibody levels above the seroprotective threshold for diphtheria, tetanus, and poliovirus types 1 and 2, and 99.3% of participants (all but one) were seroprotected against poliovirus type 3 (Table 2). Against Hib, 179 (98.4%) participants had anti-PRP antibody concentrations ≥0.15 µg/mL. At least 98.9% of participants were seropositive for each of the three pertussis antigens (Table 3).

**Table 2.** Seroprotection rates and antibody GMCs/GMTs post-primary and post-booster vaccination against diphtheria, tetanus, *H. influenzae* type b, and poliovirus 1–3 antigens (ATP cohorts).

	Threshold	Time point	N	Seroprotection rate (95% CI)	GMC/GMT (95% CI)
Anti-diphtheria	0.1 IU/mL	Post-primary	176	100.0 (97.9–100.0)	3.24 (2.84–3.68)
	0.1 IU/mL	Post-booster	188	100.0 (98.1–100.0)	12.11 (10.82–13.56)
	1 IU/mL		187	99.5 (97.1–100.0)	
Anti-tetanus	0.1 IU/mL	Post-primary	176	100.0 (97.9–100.0)	3.14 (2.81–3.51)
	0.1 IU/mL	Post-booster	188	100.0 (98.1–100.0)	8.18 (7.35–9.11)
	1 IU/mL		188	100.0 (98.1–100.0)	
Anti-PRP	0.15 µg/mL	Post-primary	179	98.4 (95.3–99.7)	2.97 (2.48–3.54)
	0.15 µg/mL	Post-booster	188	100.0 (98.1–100.0)	28.72 (24.70–33.40)
	1.0 µg/mL		188	100.0 (98.1–100.0)	
Anti-poliovirus 1	8 ED_50_	Post-primary	151	100.0 (97.6–100.0)	613.9 (505.5–745.5)
		Post-booster	176	100.0 (97.9–100.0)	2185.4 (1901.1–2512.3)
Anti-poliovirus 2	8 ED_50_	Post-primary	151	100.0 (97.6–100.0)	591.6 (487.3–718.3)
		Post-booster	169	100.0 (97.8–100.0)	2944.1 (2601.3–3332.2)
Anti-poliovirus 3	8 ED_50_	Post-primary	151	99.3 (96.4–100.0)	827.4 (674.7–1014.6)
		Post-booster	167	100.0 (97.8–100.0)	3684.6 (3225.3–4209.3)

ATP, according-to-protocol; GMC, geometric mean concentration; GMT, geometric mean titer; N, number of participants with available results; CI, confidence interval; IU, International Units; Post-primary, one month after the primary course of vaccination; Post-booster, one month after the booster vaccine dose; PRP, polyribosyl-ribitol phosphate; ED_50_, median effective dose.

Assay cutoffs were 0.057 IU/mL (diphtheria), 0.043 IU/mL (tetanus), 0.066 µg/mL (anti-PRP), and a titer ≥8 (poliovirus 1–3).

**Table 3.** Seropositivity rates and antibody GMCs post-primary and post-booster vaccination against pertussis antigens (ATP cohorts).

	Assay cutoff	Time point	N	Seropositivity rate (95% CI)	GMC (95% CI)
Anti-PT	2.693 IU/mL	Post-primary	176	98.9 (96.0–99.9)	65.0 (57.7–73.2)
		Post-booster	188	100.0 (98.1–100.0)	107.9 (96.5–120.7)
Anti-FHA	2.046 IU/mL	Post-primary	176	99.4 (96.9–100.0)	120.2 (107.0–135.1)
		Post-booster	188	100.0 (98.1–100.0)	268.4 (242.4–297.2)
Anti-PRN	2.187 IU/mL	Post-primary	176	99.4 (96.9–100.0)	166.1 (146.8–187.8)
		Post-booster	187	100.0 (98.0–100.0)	563.4 (495.6–640.5)

ATP, according-to-protocol; GMC, geometric mean concentration; N, number of participants with available results; CI, confidence interval; PT, pertussis toxoid; Post-primary, one month after the primary course of vaccination; Post-booster, one month after the booster vaccine dose; FHA, filamentous hemagglutinin; PRN, pertactin.

Seropositivity rates were defined as the percentage of participants with antibody concentrations above the assay cutoff.

One month after the booster vaccination, all infants had seroprotective antibody levels against diphtheria, tetanus, and poliovirus types 1–3. All participants had anti-PRP antibody concentrations ≥1.0 µg/mL and were seropositive for each of the PT, FHA and PRN antigens.

Between primary and booster vaccinations, considerable increases in antibody concentrations and titers were observed for all vaccine antigens (Tables 2, 3).

### Safety and reactogenicity

**Figure 2.**
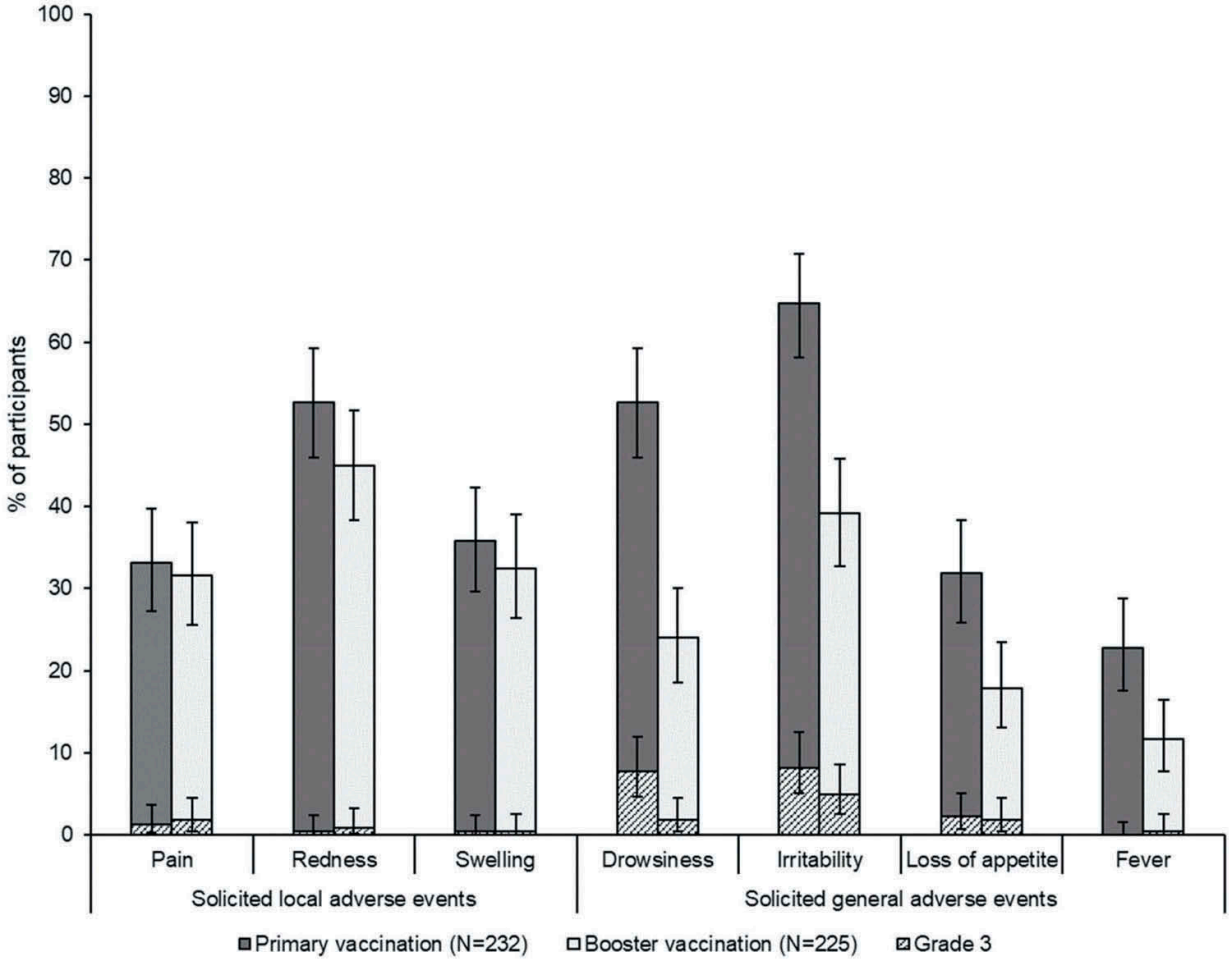
Solicited adverse events (total vaccinated cohorts) N, number of participants for primary and booster vaccination course with at least one documented dose.

The most commonly reported local AE was redness after both primary (52.6%) and booster (44.9%) vaccinations. Irritability was the most frequent general AE, reported for 64.7% of children following the primary and 39.1% following the booster vaccination; any fever was recorded for 22.8% and 11.6% of children, respectively (Figure 2). The most common local AE of grade 3 intensity was pain, reported by 3 (1.3%) infants after the primary and 4 (1.8%) toddlers after the booster vaccination. Grade 3 irritability was reported by 19 (8.2%) participants following the primary and 11 (4.9%) participants following the booster vaccination. One child experienced grade 3 fever after the booster dose, that was considered vaccination-related (Figure 2).

One child experienced a large swelling reaction the day after the booster vaccination, with a maximum diameter of 80 mm. The swelling resolved within seven days.

During the 30-day post-vaccination period in the primary phase, at least one unsolicited AE was reported for 48 (20.4%) infants; 2 (0.9%) infants experienced an unsolicited AE considered vaccination-related (agitation and erythema) (Table 4). One (0.4%) infant experienced a grade 3 unsolicited AE (rhinitis) considered unrelated to vaccination by the investigator. During the 30-day post-booster period, unsolicited AEs were recorded for 13 (5.8%) children; for one child (0.4%), one event (nightmare) was assessed by the investigator to be vaccination-related. No grade 3 unsolicited AEs were reported in the 30-day period after the booster dose. Medically attended AEs were recorded for 23 (9.8%) participants after the primary and 6 (2.7%) participants after booster vaccination (Table 4).

**Table 4.** Percentage of participants with reported unsolicited adverse events and serious adverse events (total vaccinated cohorts).

	Primary vaccination (N = 235)		Booster vaccination (N = 225)	
	n	% (95% CI)	n	% (95% CI)
Any unsolicited AEs	48	20.4 (15.5–26.2)	13	5.8 (3.1–9.7)
Grade 3	1	0.4 (0.0–2.3)	0	0.0
Related to vaccination	2	0.9 (0.1–3.0)	1	0.4 (0.0–2.5)
Medically attended AEs	23	9.8 (6.3–14.3)	6	2.7 (1.0–5.7)
SAEs up to study end, n (%)				

N, number of participants; n (%), number (percentage) of participants reporting at least one AE; AE, adverse event; CI, confidence interval; SAE, serious AE.

A total of six SAEs were reported for three (1.3%) infants: one infant experienced gastric infection, one infant experienced anal fistula and proctitis, and one infant experienced a circulatory collapse, congenital heart disease, and patent ductus arteriosus. All SAEs were considered by the investigator as unrelated to vaccination and all infants recovered by study end. No fatalities were reported during the study.

## Discussion

The combined DTPa-IPV/Hib vaccine induced robust immune responses to all vaccine antigens after three primary doses and a booster dose and had an acceptable safety profile when administered to healthy Russian children before their second year of life. A booster effect on antibody concentrations was observed for all vaccine antigens.

All participants achieved seroprotective levels against diphtheria and tetanus one month after the primary and booster vaccinations. This is in line with data from previous studies conducted in healthy children in Asia and Europe.^15,29,30^ When infants received DTPa-IPV and Hib vaccines, administered separately or combined as a single injection, according to a 2-4-6-months primary schedule followed by a booster dose at 16–19 months of age, seroprotection rates for diphtheria and tetanus were 100% one month after the primary series, and declined in time, but returned to 100% one month after the booster dose.^13^ In another study evaluating the immune responses of DTPa-IPV/Hib co-administered with a rotavirus vaccine, 97.3% of participants were seroprotected against diphtheria and 100% against tetanus after three vaccine doses administered at 3, 4 and 5 months of age.^17^

In the present study, 98.9% of participants were seropositive for PT and 99.4% for FHA and PRN antigens one month after the primary vaccination. Similar results have been observed in trials conducted in European infants, comparing the immune responses to pertussis vaccination following vaccination with either an HBV-containing hexavalent combination (DTPa-HBV-IPV/Hib) or the DTPa-IPV/Hib and HBV vaccines separately.^29,31^ In several studies conducted in Asian infants, seropositivity rates of 100% were also reported for all three pertussis antigens following primary vaccination with DTPa-IPV/Hib according to different schedules.^14,15,30^ In the current study, at one month after the booster dose, all participants were seropositive for all three pertussis antigens, in line with previous reports.^15,30^ As different assays and seropositivity thresholds were used across studies, seropositivity rates cannot be directly compared. However, data indicate the mounting of robust immune responses against pertussis antigens following vaccination with DTPa-IPV/Hib.

Almost all children (98.4%) achieved anti-PRP antibody levels ≥0.15 µg/mL one month following the primary vaccination. While several studies reported seroprotective levels for 100% of study participants after completing the primary vaccination course,^17,29,30^ in other reports seroprotective rates ranged between 96.4% and 98.7%.^13,15^ One month post-booster dose, all study participants achieved seroprotective levels indicative of long-term protection (≥1.0 µg/mL) against Hib. In other studies, lower anti-PRP antibody levels were observed in children receiving the combined DTPa-IPV/Hib vaccine as compared with those who received the Hib vaccine as a separate injection.^13,14,16^ Nevertheless, the lower anti-PRP antibody responses to combined DTP-Hib vaccines were previously shown not to be associated with an impaired function of the induced antibodies, nor with impaired immune memory against Hib.^32,33^ The successful long-term Hib disease control in Europe achieved with a DTPa-HBV-IPV/Hib combination vaccine also confirms that these immunological findings have no clinical impact.^34^

The immune responses observed in the current study against poliovirus types 1–3 one month following the primary vaccination were in line with previous reports. In Caucasian infants who completed a 3-dose primary schedule with either DTPa-HBV-IPV/Hib or DTPa-IPV/Hib + HBV vaccines, seroprotection rates were achieved by almost all participants; antibody levels ranged between 481.6–1590.3 ED_50_ (poliovirus 1), 350.6–1961.2 ED_50_ (poliovirus 2), and 1152.3–2425.5 ED_50_ (poliovirus 3).^29,35^

The three primary doses of DTPa-IPV/Hib vaccine administered at 3, 4.5, and 6 months of age and the booster dose administered at 18 months of age were well tolerated, with an acceptable reactogenicity profile. The frequencies of the observed solicited local and general AEs were similar to those reported from other studies, with redness and irritability being the most frequent solicited local and general AEs (of all grades), respectively, although the vaccination schedules differed across studies.^14,29,30,36^ In line with previous reports,^15,37^ the most commonly reported grade 3 local and general solicited AEs were pain and irritability, respectively. While the incidences of solicited local AEs remained similar, the incidence of solicited general AEs in the current study tended to be lower after the booster dose as compared with primary vaccination. The frequencies of AEs observed following booster vaccination were nevertheless similar with those reported from a phase 3 trial evaluating the safety of the booster dose of DTPa-IPV/Hib in Vietnamese toddlers.^12^

Consistent with previous reports,^14,15^ the occurrence of vaccine-related unsolicited AEs was not frequent. No SAEs that occurred during the study were considered related to the study vaccine. The observed 0.4% frequency of large swelling reactions is comparable with literature data.^36,37^

The strengths of this study included the successful enrollment of a large number of children from 5 different sites distributed over the Russian Federation, the laboratory testing conducted in one central laboratory, and the use of validated laboratory tests. The open-label and non-randomized design might be one of the limitations of the study. The number of evaluable participants for the primary endpoint of the study on immunogenicity was not derived from a sample size powered computation but based on the required number of participants as requested by the Russian Regulatory Authorities. The trial was conducted in one country, though the results could be generalized to other populations with similar disease prevalence and immunization practices. The lack of data on pre-primary and pre-booster immune responses in infancy did not allow the assessment of the fold-change of antibody levels from pre – to post-vaccination for the primary and booster immunization series. Co-administration of DTPa-IPV/Hib with an HBV vaccine was not envisaged in the study protocol, however, concomitant administration with HBV or any other vaccine as part of the national immunization schedule and as part of routine vaccination practice was allowed. Additionally, broad literature data exist to support the concomitant injection of DTPa-IPV/Hib and HBV vaccines^30,35,38,39^ and many countries use these vaccines as standard of care.

The coverage of pediatric DTP immunization in the Russian Federation has remained high for more than a decade, with 97% of children receiving the third DTP dose.^40,41^ However, resurgences of childhood diseases still occur. Therefore, a high uptake of pediatric vaccinations remains paramount and the administration of combination vaccines has been shown to improve vaccination compliance.^10^ Moreover, in the Russian Federation whole-cell pertussis vaccination predominates over the acellular pertussis vaccines.^42^ The use of an acellular pertussis containing combination vaccine like DTPa-IPV/Hib might therefore contribute to improved vaccination acceptance, as this vaccine is less reactogenic than whole-cell pertussis vaccines, enhancing compliance and coverage.^43^

**Figure 3.**
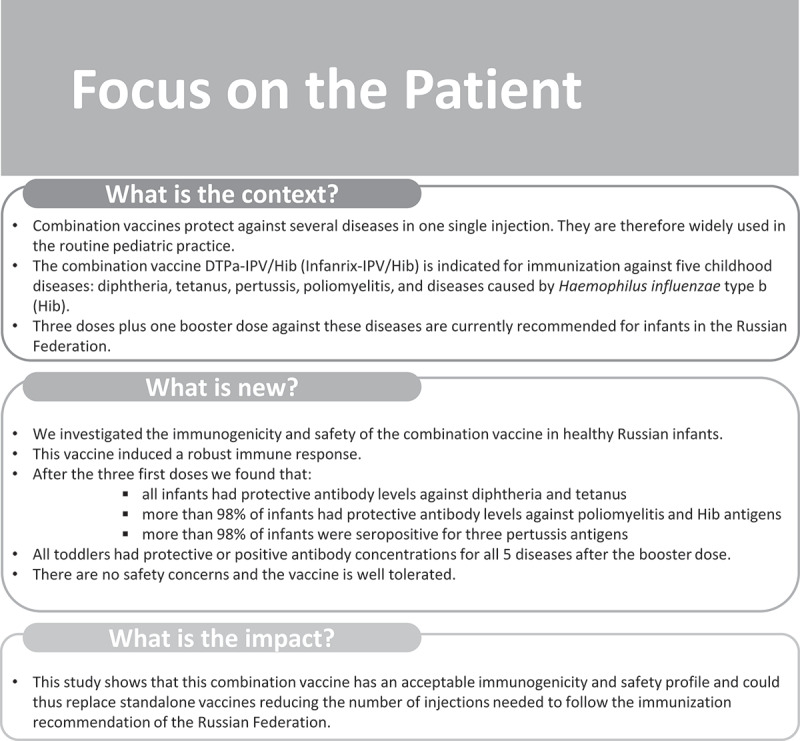
Plain language summary.

In conclusion, the combined DTPa-IPV/Hib vaccine administered as a 3-dose primary vaccination at 3, 4.5 and 6 months of age and a booster dose at 18 months of age induced robust immune responses to all vaccine antigens and was well tolerated in healthy Russian infants. For the benefit of healthcare professionals, a summary contextualizing the results and relevance of this clinical research is displayed in the Focus on Patient Section (Figure 3).

## Supplementary Material

Supplemental Material

## Abbreviations

AE: adverse event
ATP: according-to-protocol
CI: confidence interval
DTP: diphtheria-tetanus-pertussis
DTPa-IPV/Hib: diphtheria-tetanus-acellular pertussis-inactivated polio and *Haemophilus influenzae* type b conjugate vaccine
DU: D-antigen units
ED_50_: median effective dose
FHA: filamentous hemagglutinin
GMC: geometric mean concentration
GMT: geometric mean titer; HBV, hepatitis B virus
Hib: *Haemophilus influenzae* type b
IU: International Units; PRN, pertactin
PRP: polyribosyl-ribitol phosphate
PT: pertussis toxoid
SAE: serious adverse event

## Data sharing statement

The results summary for this study (GSK study number 116194, gcp02858440) is available on the GSK Clinical Study Register and can be accessed at www.gsk-clinicalstudyregister.com.

For interventional studies that evaluate our medicines, anonymized patient-level data will be made available to independent researchers, subject to review by an independent panel, at www.clinicalstudydatarequest.com within six months of publication.

To protect the privacy of patients and individuals involved in our studies, GSK does not publicly disclose patient-level data.

## Trademark statement

*Infanrix-IPV/Hib* is a trademark of the GSK group of companies.
