# Supplementary material for: Immunogenicity and safety of a combined DTPa-IPV/Hib vaccine administered as a three-dose primary vaccination course and a booster dose in healthy children in Russia: a phase III, non-randomized, open-label study
Source: Hum Vaccin Immunother. 2020 Feb 12;16(9):2265–73. doi: 10.1080/21645515.2020.1720437 (PMC7553713; doi:10.1080/21645515.2020.1720437)
Supplement: Supplemental Material [file KHVI_A_1720437_SM0586.docx]

**Supplemental material**

**Supplemental text.** Inclusion and exclusion criteria

All children must have satisfied all of the following criteria for enrollment:

- Children’s parent(s)/adoptive parent(s) who, in the opinion of the investigator, could and would comply with the requirements of the protocol;
- Male or female child between 3 and 4 months (between, and including, 90 and 120 days) of age at the time of the first vaccination;
- Written informed consent obtained from the parent(s)/adoptive parent(s) of the child prior to performing any study specific procedure;
- Healthy children as established by medical history and clinical examination before entering into the study;
- Born full-term (i.e. after a gestation period of at least 37 completed weeks).

Children were not eligible for enrollment if they met any of the following criteria:

- Placed under the control or protection of an agency, organization, institution or entity by the courts, the government or a government body, acting in accordance with powers conferred on them by law or regulation (Not including adopted children or those with an appointed legal guardian);
- Use of any investigational or non-registered product (drug or vaccine) other than the study vaccine during the period starting 30 days before the first dose of study vaccine, or planned use during the study period;
- Any medical condition that in the judgment of the investigator would have made intramuscular injection unsafe;
- Chronic administration (defined as more than 14 days in total) of immunosuppressants or other immune-modifying drugs during the period starting since birth. For corticosteroids, this meant prednisone ≥0.5 mg/kg/day (for pediatric participants), or equivalent. Inhaled and topical steroids were allowed;
- Administration of long-acting immune-modifying drugs at any time during the study period;
- Planned administration/administration of a vaccine not foreseen by the study protocol in the period starting 30 days before the first dose and ending 30 days after the last dose of vaccine administration, with the exception of hepatitis B and other vaccines given as part of the national immunization schedule and as part of routine vaccination practice, that are allowed at any time during the study period. Seasonal or pandemic influenza vaccine could have been given at any time during the study, and according to the Summary of Product Characteristics and national recommendations;
- Concurrent participation in another clinical study, at any time during the study period, in which the child was exposed to an investigational or a non-investigational vaccine/product (pharmaceutical product or device);
- Previous vaccination against diphtheria, tetanus, pertussis, poliomyelitis and *Haemophilus influenzae* type b (Hib) diseases;
- History of diphtheria, tetanus, pertussis, poliomyelitis and Hib diseases;
- Any confirmed or suspected immunosuppressive or immunodeficient condition, based on medical history and physical examination (no laboratory testing required);
- Family history of congenital or hereditary immunodeficiency;
- History of any reaction or hypersensitivity likely to have been exacerbated by any component of the vaccine;
- Major congenital defects;
- Serious chronic illness;
- History of any neurological disorders or seizures;
- Acute disease and/or fever at enrolment (Fever was defined as temperature ≥37.5°C for oral, axillary or tympanic route, or ≥38.0°C for rectal route). Children with a minor illness (such as mild diarrhea, mild upper respiratory infection) without fever could have been enrolled at the discretion of the investigator;
- Administration of immunoglobulins and/or any blood products since birth or planned administration during the study period.
